# Serum osteocalcin levels in girls with central precocious puberty

**DOI:** 10.1186/1687-9856-2013-S1-P79

**Published:** 2013-10-03

**Authors:** Young Jun Rhie, Hyo Kyoung Nam, Kee Hyoung Lee

**Affiliations:** 1 Department of Pediatrics, Korea University College of Medicine, Seoul, South Korea

## Objectives

Bone plays metabolic roles through osteocalcin(OC) when it is released into the systemic circulation in uncarboxylated form. Identified novel metabolic roles of OC include increasing insulin secretion and sensitivity, energy expenditure, reduction of fat mass and mitochondrial proliferation and functional enhancement. The onset of puberty can be influenced metabolic factors. This study was aimed to determine serum OC levels in girls with central precocious puberty(CPP) and to investigate the effects of OC on the onset of puberty.

## Methods

Serum OC levels of girls CPP (n=30) and their age-matched controls (n=30) were measured. GnRH stimulation test was performed in CPP group. Bone age was determined in all subjects.

## Results

Serum OC levels were significantly higher in CPP group compared with control group (76.8 ± 10.5 vs. 61.6 ± 15.1ng/mL, p=0.001). Serum OC levels were correlated with peak LH levels during GnRH stimulation test (r=0.348, p=0.037), bone age (r=0.403, p=0.010) and bone age advance (r=0.323, p=0.042), but not related to age, height, weight and BMI.

## Conclusions

Serum OC seems to be associated with the onset of puberty leaving casual relations unresolved.

